# Seasonal Growth Rate of the Sponge *Haliclona oculata* (Demospongiae: Haplosclerida)

**DOI:** 10.1007/s10126-008-9086-9

**Published:** 2008-02-22

**Authors:** Marieke Koopmans, René H. Wijffels

**Affiliations:** Department of Agrotechnology and Food Sciences, Bioprocess Engineering Group, Wageningen University, 6700 EV Wageningen, The Netherlands

**Keywords:** Haliclona oculata, Growth rate, Nutrients, Oosterschelde, Photogrammetry, Sponge biotechnology

## Abstract

The interest in sponges has increased rapidly since the discovery of potential new pharmaceutical compounds produced by many sponges. A good method to produce these compounds by using aquaculture of sponges is not yet available, because there is insufficient knowledge about the nutritional needs of sponges. To gain more insight in the nutritional needs for growth, we studied the growth rate of *Haliclona oculata* in its natural environment and monitored environmental parameters in parallel. A stereo photogrammetry approach was used for measuring growth rates. Stereo pictures were taken and used to measure volumetric changes monthly during 1 year. Volumetric growth rate of *Haliclona oculata* showed a seasonal trend with the highest average specific growth rate measured in May: 0.012 ± 0.004 day^−1^. In our study a strong positive correlation (*p* < 0.01) was found for growth rate with temperature, algal biomass (measured as chlorophyll a), and carbon and nitrogen content in suspended particulate matter. A negative correlation (*p* < 0.05) was found for growth rate with salinity, ammonium, nitrate, nitrite, and phosphate. No correlation was found with dissolved organic carbon, suggesting that *Haliclona oculata* is more dependent on particulate organic carbon.

## Introduction

Since the discovery of pharmaceutical interesting compounds in sponges many attempts have been made to culture sponges for the production of these metabolites. Sponges are sessile benthic organisms that are able to change their shape and internal structure to cope with environmental changes (Simpson 1984; Garrabou and Zabala 2001; Mendola et al. 2007). Despite the ability of sponges to adjust to changes in their environment only a low number of successful ex situ culture systems have been developed, although very low and variable growth rates were obtained in these systems (de Caralt et al. 2003; Mendola 2003; Duckworth and Pomponi 2005; Sipkema et al. 2005). So far sponges cultured in their natural habitat gave the best results especially for long-term survival. However, a disadvantage of this method is that conditions cannot be controlled or optimized.

Many different factors can cause variability in sponge growth, such as size, age, damage, and environmental conditions (Ayling 1983; Handley et al. 2003). The effect of these factors on sponge growth and development is not well understood. By studying sponge growth rates in their natural environment a better understanding can be obtained. In the field seasonal trends have been found in growth rates. Several researchers (Barthel 1986; Garrabou and Zabala 2001; Page et al. 2005; de Caralt et al. 2007) found a positive relationship between temperature and growth rate. Besides temperature, nutrient availability affects growth (Duckworth and Pomponi 2005). Optimal conditions can be applied to laboratory cultures to obtain continuous cultures that are not affected by seasonal growth patterns.

Preferred conditions for sponge culture can be deduced by determining the highest growth rate observed while simultaneously measuring nutrient concentrations, algal biomass, and temperature. The role of dissolved organic carbon (DOC) as a food source for sponges is not well understood. Yahel et al. (2003) found that most of the carbon retained by sponges came from the dissolved pool. Particulate organic carbon (POC) was found to be the food source for *Dysidea avara* (Ribes et al. 1999). Studying shifts in both particulate organic carbon and dissolved organic carbon will provide more insight in the relative contribution of POC and DOC for the growth of sponges.

Many of the methods used for measuring growth are destructive, such as determining underwater weight, wet weight, and ash-free dry weight. Photographing and measuring sponge surface area is nondestructive and provides good results for thinly encrusting sponges (Garrabou and Zabala 2001; de Caralt et al. 2007). For massive and branching sponges surface area determination will not be accurate enough because they grow in three dimensions. Abdo et al. (2006) developed a method to nondestructively measure volume of sessile benthic organisms by using a stereo photogrammetry method. Stereo photogrammetry simulates depth by simultaneously displaying two overlapping images. They have shown that the method is accurate and takes short data processing and analysis time. Using this method, it is possible to monitor volumetric growth rates long-term for sessile organisms without disturbing the individuals.

We used the branching sponge *Haliclona oculata* for measuring growth rate. Many different bioactive compounds have been found in the *Haliclona* order, such as lectins, peptides, ketosteroids, and sterol esters (Pajic et al. 2002; Aoki et al. 2003; Santalova et al. 2003). Also in *Haliclona oculata* from various places compounds like sterols were found (Findlay and Patil 1984; Yu et al. 2006). For *Haliclona oculata* from the Netherlands no information is available about production of bioactive compounds. Our main interest was studying sponge growth rate in general, and *Haliclona oculata* is one of the most abundant sponges in the Netherlands; therefore, this species was chosen as the model sponge in our research.

In this study, we monitored growth of *Haliclona oculata* monthly in the Oosterschelde in the Netherlands from September 2005 to September 2006 using a stereo photogrammetry technique. No previous studies have been reported on nondestructive measuring the volumetric growth rate of sponges using three-dimensional photogrammetry. Apart from growth rate, food availability, temperature and salinity changes are measured to find possible relationships between these factors and sponge growth rate.

## Material and Methods

### Study Area

**Fig. 1:**
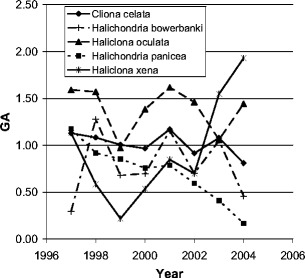
General abundance of different species of sponges at Lokkersnol, Oosterschelde, the Netherlands. GA is a logarithmic scale in the range of 0 to 3, 1 means that, on average, during every dive 1 individual of this species was found, 2 stands for 10 individuals per dive, and 3 for 100. Data obtained from Stichting ANEMOON and are partly published in Gmelig Meyling and de Bruyne 2003

*Haliclona oculata* was monitored in the Netherlands, Oosterschelde estuary (Lokkersnol, 51°38′58.07″N, 3°53′5.11″E) at 13 m depth. *H. oculata* widely occurs in the Oosterschelde and grows attached to oyster shells. According to monitoring data of the Dutch ANEMOON foundation, *H. oculata* was the most stable sponge present at the dive site Lokkersnol for at least 8 years (Fig. 1). General abundance (GA) in Fig. 1 is a logarithmic value and stands for general abundance of a certain species. *Cliona celata* was stable in specimen number present as well, but this sponge is impossible to monitor in volumetric changes because it is an excavating sponge. Both *Halichondria panicea* and *Halichondria bowerbanki* were decreasing in number throughout the Oosterschelde during the last 8 years. *Haliclona xena* was increasing in number during the last years but has an annual cycle, and it disappeared at the start of our monitoring period.

**Fig. 2:**
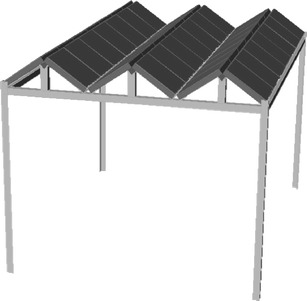
Platform that was placed at the study site containing 42 tiles, sponges were attached on every tile. Size platform: 2.5-m × 1.6-m × 1.2-m

A platform (Fig. 2) was placed at the study site and 42 concrete pavement tiles (30-cm x 30-cm) were placed on top of the platform. On every tile a sponge specimen was attached using elastic bands. Specimens were attaching to the tiles within 1 or 2 months. For 1 year, from September 2005 to September 2006, sponge growth was monitored.

### Monitoring Growth

**Fig. 3:**
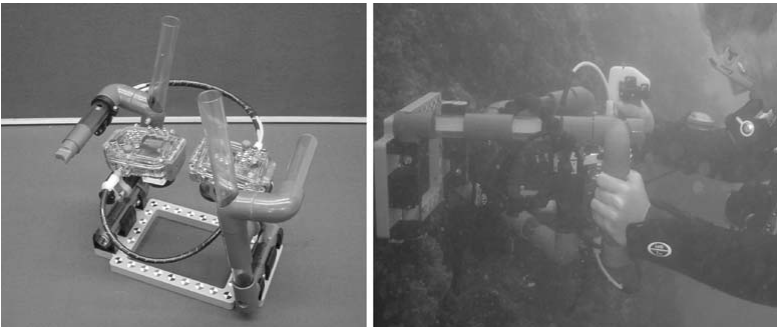
Stereo camera system. Left image shows a top view of the system, and right image shows the system being used under water

Monthly, pictures were taken using a stereo camera system (Fig. 3) to monitor volume changes in time. The camera system consisted of two Nikon coolpix 5200 five megapixel digital cameras. The two cameras were mechanically connected to make pictures simultaneously. Both cameras were set on autofocus, macro, iso 100, no flash, and no zoom. A twin set 50W video lights was mounted to the camera system to provide light at low light conditions. A reference frame was connected to the camera frame, which ensured pictures to be taken at the same distance every time. Reference points were present on the frame for calibration.

Calibration of the camera system was performed in two steps. The first step of calibration used a photogrammetric bundle adjustment to determine camera calibration properties (focal length, principal point location, radial and tangential distortion). The calibration was performed using imagery of a calibration fixture captured underwater (see also Abdo et al. 2006). This first step was only done once for the camera system. Because the camera positions at the different monitoring dates were not exactly similar, a second step of calibration was necessary.

The second step was needed to adjust the camera calibration properties according to the coordinated reference frame visible in all stereo images. Using the properties of the first calibration, measurements were made on the coordinated reference frame. These measured values were used to determine whether large deviations were found and camera properties and relative orientation of the cameras needed to be recomputed.

### Measure Volume

**Fig. 4:**
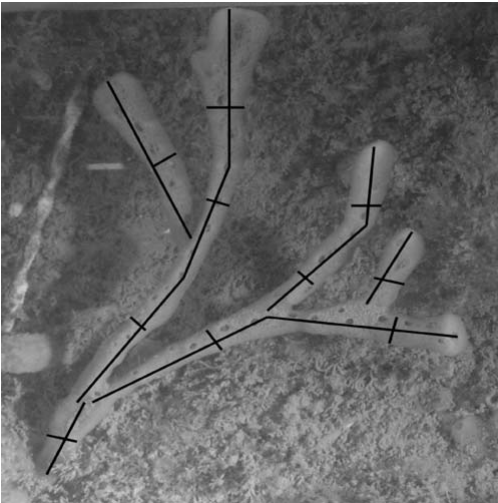
Illustration of computed lengths in one image of a stereo pair

*Haliclona oculata* is a branching sponge, which makes three-dimensional reconstructions impossible, because branches were overlapping in pictures from different views. Therefore, we reconstructed the branches as cylinders. In all stereo images, length and width was determined for all branches. Lengths were measured using stereo calibration and measurement software (CAL and PhotoMeasure) created by J. Seager (http://www.seagis.com.au). In one stereo pair, start and end of a branch was indicated and the program determined branch length. Figure 4 shows how the lengths and widths of the sponge branches were measured.

For every specimen and time point, minimally three stereo images were made. In these three stereo images, all lengths and widths were measured and averages were used in further calculations. Volume was determined by using the following equation:

$$\left. {V = \sum {V_n \Rightarrow V_n = 0.25 \cdot \pi \cdot \left( {Average\left( {W_n } \right)} \right)^2 \cdot Average} \left( {L_n } \right)} \right)\;\;\;\;\;\;\left[ {{\text{mm}}^{\text{3}} } \right]$$

V_n_, W_n_ and L_n_ are volume, width and length of branch n, respectively. Total volume of the sponge could not be measured in every stereo pair; therefore, lengths and widths were averaged instead of total volume.

Growth rates between two data points were determined exponentially by using the following equation:

$$\mu _t = \frac{{Ln\left( {\frac{{V_t }}{{V_{t - 1} }}} \right)}}{{dt}}\;\;\;\;\;\;\left[ {{\text{day}}^{ - {\text{1}}} } \right]$$

Here, μ_t_ is specific growth rate at time t, V_t_ is the volume at time t, V_t-1_ is the volume at time t - 1 month, and dt is the time between two measurement points in days. Because circumstances change continuously, specific growth rate was determined per time interval.

### Volume Versus Ash-free Dry Weight

To determine whether the calculated volumes were representative for the amount of biomass, eight individuals were used to make a calibration curve. Different sized sponges were used and pictures were taken under water for volume measurement. Afterward the sponges were dried in a stove overnight at 80°C. Dry weight was determined and before the sponges were burned at 450°C for 4 hr to determine their ash-free dry weight.

### Nutrient Availability

The Netherlands Institute of Ecology monitored monthly and from April to September twice per month the nutrient and pigment concentration in the Oosterschelde Estuary (51°37′12.94″N and 3°55′19.85″E). Samples were taken from surface water, which is representative because the Oosterschelde is well mixed due to the regular tide. Analyses of nitrite, nitrate, phosphate, and dissolved organic carbon (DOC) were performed by using a Skalar auto analyser (see also Goosen et al. 1995).

Water samples were filtered by using a GF/F filter (0.7 μm) and suspended particulate matter (SPM) was determined by weighing. Total nitrogen and organic carbon was analyzed in SPM in a Carlo-Erba NA-1500 elemental analyser according to Nieuwenhuize et al. (1994). Plant pigments were extracted from SPM samples using 95% methanol buffered with 5% ammonium acetate according to the method used by Barranguet et al. (1997) and after centrifugation chlorophyll a was analyzed by HPLC. Temperature and salinity were monitored during the monthly survey as well.

Nutrients and pigments were not measured at the same days as sponge volume was measured. As we have calculated growth rate from time t-1 to time t, the nutrient and pigment concentration was averaged for the period of time t-1 to time t or the value measured halfway this period was used.

### Statistical Methods

Significant differences in growth rate between different time intervals were analyzed by one-way ANOVA. The Tukey test was used for pair comparisons. Homogeneity assumption is justified; this was tested by calculation of the Levene statistic (SPSS® 12.0.1, Inc., 1989–2003).

Pearson correlation coefficient was used to find relationships between water temperature, salinity, nutrient concentration, pigment concentration, and growth rate (SPSS® 12.0.1).

## Results

### Growth

**Fig. 5:**
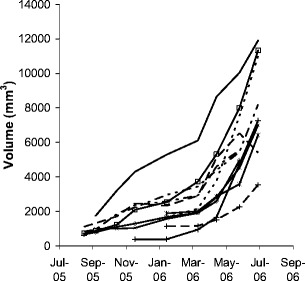
Time series of measured volumes using stereo photogrammetry of 11 monitored specimen of *Haliclona oculata* in the Oosterschelde, the Netherlands

All specimens included in this study show an increase in volume for every time interval measured, except for one individual that got partly consumed by a predator during the last measurement interval (Fig. 5). Eleven of 42 individuals were monitored for a long period and included in this study. The others did not attach or were damaged from predation or insufficient pictures of good quality were available. Massive mortality occurred in August to September 2006, and all specimens of the study died. Thus, we were forced to stop the monitoring. *Haliclona oculata* specimens around the platform died in the same period; thus, it was a natural event.

**Fig. 6:**
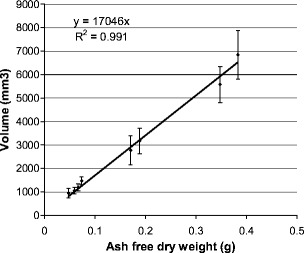
Calibration curve of measured volume using stereo photogrammetry and ash-free dry weight of *Haliclona oculata*

The volumes measured using photomeasure are compared with ash-free dry weight (Fig. 6). A good linear correlation (r^2^ = 0.991) is found between dry weight and the volume measurement demonstrating that the volume measurement as performed in this study is representative for the amount of biomass and thus can be used to calculate growth rate.

### Growth Rate

**Fig. 7:**
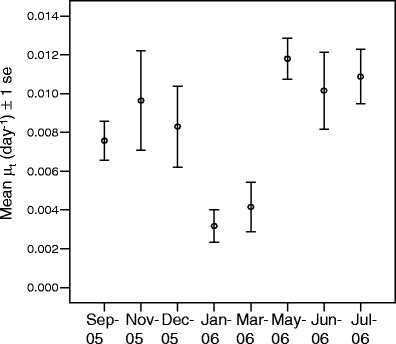
Time series of average growth rate per day with its standard error for *Haliclona oculata* in the Oosterschelde, Netherlands

Both total branch length and average branch width increased in time. Therefore, we assume exponential growth instead of linear growth. Figure 7 shows the average growth rate at the different time intervals. Growth rate shows a temporal pattern. The maximum average specific growth rate measured was 0.0118 ± 0.0035 day^−1^ (average μ ± SD), which was measured at the beginning of May 2006. The lowest average growth rate was recorded in the end of January and was 0.0032 ± 0.0025 day^−1^. The average annual growth rate was 0.0082 ± 0.0055 day^−1^.

Figure 7 shows that there is a variation in the measured specific growth rates. Therefore, a one-way ANOVA test was used to find significant differences between growth rates measured on the different dates. This showed a significant difference between at least two groups (*p* < 0.01). Using a post hoc Tukey test, a significant difference was found between growth rates in winter (January and March) and spring (May, June, and July) (*p* < 0.01). This difference might be due to changes in circumstances. Growth rates in September, November, and December are close to the overall average growth rate, and no significant difference could be found for these months and other months.

### Nutrient Availability

**Fig. 8:**
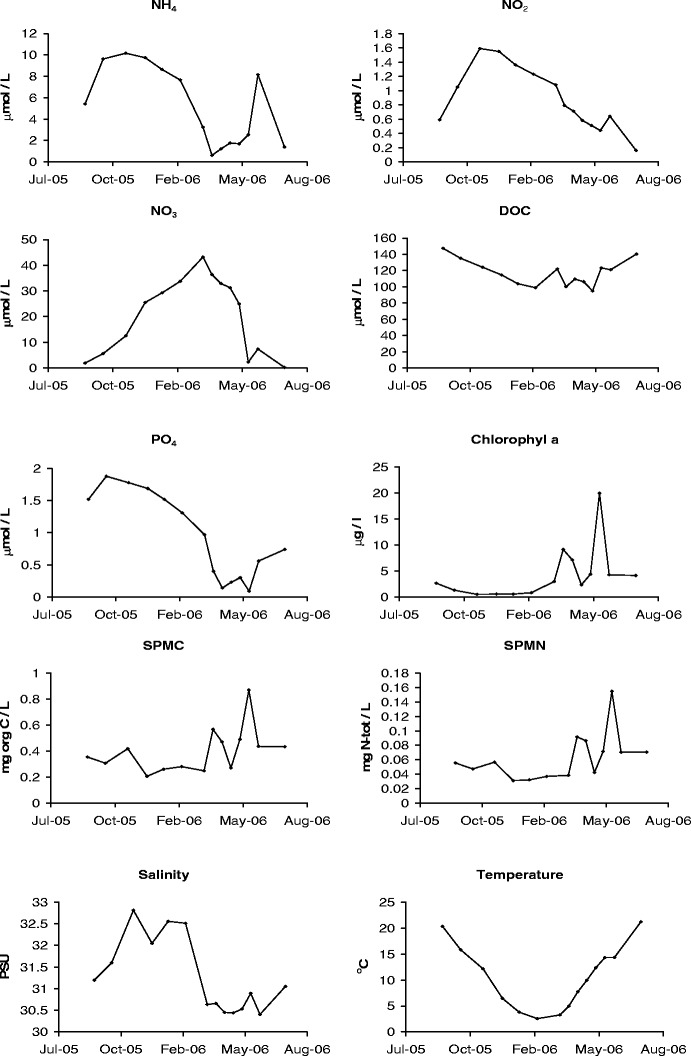
Time series of nutrients, pigments, salinity, and temperature. SPMC and SPMN stand for carbon and nitrogen content in suspended particulate matter, respectively. Data obtained from Netherlands institute of ecology

Temperature, salinity, nutrient, and pigment concentration varied in time (Fig. 8). Table 1 shows the correlation coefficients and their significance level between exponential growth rate and temperature, salinity, and the different measured nutrients. A significant negative correlation with exponential growth rate is found for ammonium, nitrite, nitrate, phosphate, and salinity, whereas a significant positive correlation is found for carbon content in SPM (SPMC), nitrogen content in SPM (SPMN), water temperature, and chlorophyll a. No correlation was found between the growth rate and dissolved organic carbon (DOC), although there was a strong correlation between DOC and water temperature (*p* < 0.01; data not shown). Figure 8 shows that for most parameters a seasonal trend was found.

**Table 1:** Pearson correlation coefficients and their significance level for exponential growth rate of *H. oculata* and different nutrients and pigments concentrations in the Oosterschelde, Netherlands

Variable	NH_4_ (μmol/L)	NO_2_ (μmol/L)	NO_3_ (μmol/L)	DOC (μmol/L)	PO_4_ (μmol/L)	SPMC (mg/L)	SPMN (mg/L)	Salinity (PSU)	Temperature (°C)	Chla (μg/L)
Pearson correlation	−.263(*)	−.354(**)	−.308(*)	.183	−.319(*)	.436(**)	.451(**)	−.381(**)	.398(**)	.349(**)
N	64	64	64	64	64	64	64	64	64	64

N is the number of data points taken into account. Data used from the Dutch institute of ecology

*Correlation is significant at the 0.05 level (2-tailed)

**Correlation is significant at the 0.01 level (2-tailed)

## Discussion

Several sponge growth rate studies have been performed in the past. Table 2 shows a comparison of growth rates for different sponges. For comparison between the different measured growth rates, measurements should be done in a similar way and rates need to be expressed in the same units. In literature different ways for measuring are used, which makes it difficult to compare results. The only nondestructive method used so far was two-dimensional photography measuring two-dimensional growth rates in area (Garrabou and Zabala 2001; de Caralt et al. 2007). In this study we used a nondestructive three-dimensional photogrammetry method that seemed to be useful for measuring volumetric growth of *Haliclona oculata*. Growth rates found for *H. oculata* are in the range found for other sponges (Table 2). In most studies the linear growth rate is determined, which generates slightly higher values than the exponential growth rate. Therefore, the highest measured linear growth rate based on our data is given between brackets for comparison.

**Table 2:** Maximal specific growth rates measured for several species of sponges found in literature

Sponge species	Order	Maximal growth rate (day^−1^)	Reference
*Oscarella lobularis* (l)	*Homosclerophorida*	0.03^l^	Garrabou and Zabala 2001
*Corticium candelabrum* (e)	*Homosclerophorida*	0.018^l^	De Caralt et al. 2007 submitted
*Mycale hentscheli* (m)	*Poecilosclerida*	0.1306^l^	Page et al. 2005
*Crambe crambe* (e)	*Poecilosclerida*	0.06^l^	Garrabou and Zabala 2001
*Hemimycale columella* (m)	*Poecilosclerida*	0.035^l^	Garrabou and Zabala 2001
*Iotrochota birotulata* (b)*	*Poecilosclerida*	0.0125^e^	Wulff 1991
*Chondrosia reniformis* (m)	*Chondrosida*	0.0027^l^	Garrabou and Zabala 2001
*Aplysina fulva* (b)*	*Verongida*	0.017^e^	Wulff 1991
*Halichondria melanadocia*	*Halichondrida*	0.006^e^	Duckworth and Pomponi 2005
*Halichondria panicea* (m)	*Halichondrida*	0.028^e^	Thomassen and Riisgard 1995
*Amphimedon rubens* (b)*	*Haplosclerida*	0.0097^e^	Wulff 1991
*Haliclona permollis* (e)	*Haplosclerida*	0.023^l^	Elvin 1976
*Haliclona oculata* (b)	*Haplosclerida*	0.012^e^ (0.015^l^)	This study

*Values determined using graphs in the article

^e^Exponential growth rate

^l^Linear growth rate

Values are recalculated to get the same units; different measuring techniques are used

Growth form is indicated with l for lobate, e for encrusting, m for massive, and b for branching

*Haliclona oculata* showed only a few times negative growth, which was always related to predation. At the end of the summer, all sponges died on and around the platform and monitoring was stopped. The reason for this death is not known but could be related to the relatively high temperature of the water. Summer 2006 was a warm summer with water temperatures rising to 23°C, which are normally approximately 20°C. In other years *Haliclona oculata* survived throughout the year (data of the ANEMOON foundation).

A strong correlation was found between volumes determined using our approach and ash-free dry weight. However, the error bars calculated for growth rate show high variation. Using more individuals will give smaller confidence intervals. Another cause for the large error bars could be the assumption of cylindrical branches, which may be questionable. Due to environmental factors, such as flow, *H. oculata* can have different forms; flat like branches and thicker, more conical branches (Kaandorp 1995). In this study the sponges are exposed to the same environmental factors and the sponges showed the same growth forms, thus similar errors are made for each individual. Large error bars were also found for the volume measurements. This can be improved by changing the camera system. Cameras could only be used in auto focus. Therefore, the parameters found for the cameras after calibration were not completely equal every time. Furthermore, the images were not taken perfectly synchronically, which decreases quality. Nevertheless the correlation with ash-free dry weight was so strong (r^2^ = 0.991) that it was accurate enough to use for growth rate measurements.

Many growth studies showed that an increase in growth rate coincided with an increase in water temperature (Page et al. 2005; de Caralt et al. 2007; Garrabou and Zabala 2001; Barthel 1986). We again found a positive correlation with temperature. Besides temperature other factors change in different seasons, a strong positive correlation was found with carbon and nitrogen content in suspended particulate matter and chlorophyll a. This is to be expected as more food is available when more algae and bacteria are present in the water.

It is striking that DOC does not seem to be correlated to sponge growth rate. Yahel et al. (2003) found a strong uptake rate of DOC by sponges. The sponge species used in that study contains more than two-thirds of its biomass of bacterial symbionts, which may be responsible for the DOC uptake. *H. oculata* does not contain these high amounts of bacteria (Koopmans 2005, unpublished data), which could explain that such a relation was not found in this study. This study showed that for *H. oculata* carbon from suspended particulate matter has a correlation with growth rate. This might indicate that a sudden increase of suspended organic matter induces growth. When taking the carbon requirements for growth into account, we found that when growth rate was high, 0.0118 day^−1^, total organic carbon (SPMC + DOC) was 1.69 mgC.l^−1^ of which only 0.43 mg was SPMC. Duckworth and Pomponi (2005) found that the carbon concentration required for *Halichondria melanadocia* was 0.356 mgC.l^−1^ and Thomassen and Riisgard (1995) found 0.277 mgC.l^−1^ for *Halichondria panicea*. Growth rates for these sponges are in the same order of magnitude as for *Haliclona oculata*; this indicates that sufficient carbon is present in SPMC only. More research is necessary to find the relation between particulate and dissolved organic matter for different species of sponges.

Nutrients, such as nitrate, nitrite, ammonium, phosphate, and salinity, have a negative correlation with growth rate. Minor changes as for salinity probably has a minor effect on growth rate despite significant correlation, although not much is known about the effect of any of these nutrients on growth of sponges or other invertebrates. Ammonium was found to be lethal for marine invertebrates at a concentration of only 60 μM (Richardson and Gangolli 1993). Concentrations found in this study are somewhat lower, but they may have a negative effect on sponge growth. A nitrate concentration <320 μmol.l^−1^ is considered acceptable for marine invertebrates (Camargo et al. 2005). Almost 10-fold lower amounts are found in the Oosterschelde, which suggests that nitrate concentrations were unlikely to be deleterious for growth in this case. However, according to Muir et al. (1991) early developmental stages of some marine invertebrates well adapted to low nitrate concentrations can be susceptible to concentrations of 16 μmol.l^−1^. Nitrite levels stayed far below toxic levels found for sensitive freshwater invertebrates (Alonso and Camargo 2005).

Although it has been found that high nutrient levels can have negative effects or be lethal to marine animals, the values found in the year of our study are well below toxic levels. Nevertheless, still little is known about the effects of nutrient concentrations on growth of sponges. In in situ studies one can learn which concentrations are acceptable but finding the optimal or lethal values is difficult as many different factors change parallel to each other.

## Conclusions

Volumetric growth rate of *Haliclona oculata* could be accurately measured using a novel three-dimensional photogrammetry technique. *H. oculata* shows a seasonal trend in growth rate with an increase in growth rate when temperature and carbon and nitrogen levels in suspended particulate matter increases. No correlation is found between growth rate and dissolved organic carbon. Ammonium, nitrate, nitrite, phosphate and salinity show a negative correlation with growth rate of *H. oculata*. Growth rates measured are in the same order of magnitude as found for other sponges.
